# Fabrication and Rapid Gas-Sensing Response of NiO/ZnO p-n Heterojunctions for n-Propanol Gas

**DOI:** 10.3390/s26123655

**Published:** 2026-06-08

**Authors:** Yunfu Xing, Hongli Jia, Hongjian Liang, Yinuo Fan, Rui Zhang, Enze Ma, Ziwei Lv, Yong Tao, Xiaofeng Wang

**Affiliations:** 1 Leicester International Institute, Dalian University of Technology, Panjin 124221, China; 1783616445@mail.dlut.edu.cn (Y.X.); jiahongli145@mail.dlut.edu.cn (H.J.); fanyinuo3708021229@mail.dlut.edu.cn (Y.F.); 460941621@mail.dlut.edu.cn (R.Z.); 1273640878@mail.dlut.edu.cn (E.M.); 13512230307@163.com (Y.T.); 2 School of Physics, Dalian University of Technology, Dalian 116024, China; lianghongjian@163.com (H.L.); lvzw@mail.dlut.edu.cn (Z.L.); 3 School of General Education, Dalian University of Technology, Panjin 124221, China

**Keywords:** MOFs, p-n heterojunction, metal oxide gas sensor, random forest algorithm

## Abstract

In this study, NiO/ZnO heterojunction materials were prepared by calcining metal–organic frameworks (MOFs). The structural and morphological characteristics of the NiO/ZnO composite were investigated using various characterization methods, including X-ray diffraction, X-ray photoelectron spectroscopy, and scanning electron microscopy. Gas-sensing tests showed that at the operating temperature of 190 °C, the NiO/ZnO heterojunction (with a molar ratio of 1:1) exhibited the highest response value (*R*_a_/*R*_g_ = 201.7) and good selectivity toward 100 ppm n-propanol. Compared to pure ZnO and NiO, the response of NiO/ZnO was significantly improved (ZnO: 6, NiO: 14.6), with increases of 33.5-fold and 13.8-fold, respectively. The response and recovery times were 92 and 30 s, respectively. Additionally, to enable rapid identification of n-propanol gas concentrations, this study developed and validated a method by training and predicting response curves using a random forest algorithm, achieving identification of n-propanol gas at different concentrations (2–100 ppm) within 7 s. Finally, the enhanced sensing performance was mainly attributed to the formation of the interfacial p-n heterojunction between NiO and ZnO, together with increased surface active sites, oxygen vacancies, and chemisorbed oxygen species.

## 1. Introduction

N-Propanol is a typical volatile organic compound (VOC) widely used as an organic solvent, chemical intermediate, and cleaning agent in industries such as pharmaceuticals, printing, personal care, and petrochemicals [1,2]. Despite its widespread industrial applications, n-propanol poses a serious threat to human health and environmental safety. As a toxic and irritating VOC, long-term exposure to n-propanol can cause significant damage to the respiratory tract, skin, and central nervous system, leading to symptoms such as headaches, nausea, and eye irritation; in severe cases, it can even result in irreversible neurological damage [3]. Hence, the development of high-performance n-propanol detection technologies is of great significance for industrial environmental monitoring and occupational health protection.

Among various metal oxide semiconductor (MOS) materials, zinc oxide (ZnO), as an n-type wide-bandgap semiconductor (bandgap width of 3.4 eV), is widely used in gas-sensing research due to its high electron mobility, good chemical stability, and environmental friendliness [4]. However, pure ZnO gas sensors typically suffer from critical drawbacks such as high optimal operating temperatures (usually 300–400 °C) and poor selectivity, which severely limit their practical applications [5,6]. Similarly, nickel oxide (NiO), a typical p-type semiconductor, has also been widely used in gas-sensing research; its bandgap is generally reported to be around 3.7 eV but can vary considerably depending on oxygen-vacancy concentration and preparation conditions [7]. However, pure NiO sensors similarly suffer from low response values and high operating temperatures when detecting VOCs [8,9].

To overcome the inherent limitations of single-component MOS materials, researchers have developed various modification strategies to enhance their gas-sensing performance, including nanostructure engineering, noble metal doping, surface functionalization, and the construction of heterostructures [10,11,12]. Among these strategies, the construction of p-n or n-n heterojunctions has been regarded as an effective route for improving charge transfer and surface reaction activity. For metal oxide heterostructure sensors, the component ratio of the composite material and the concentration range of the target gas should be clearly specified, as both factors strongly affect sensing performance. ZnSnO_3_/ZnO and Zn_2_SnO_4_/SnO_2_ heterojunctions have been reported to show responses of 14.7 and 46.6 to 100 ppm n-propanol at 220 and 270 °C, respectively [13,14]. Although ZnO/NiO heterostructures have been explored for chemiresistive gas sensing, most reported systems typically rely on morphology control. For instance, Tian et al. reported that the NiO/ZnO sample with a 10% Ni content exhibited optimal ethanol-sensing performance at 200 °C [15]. Zhao et al. prepared hierarchical flower-like ZnO/NiO composites via hydrothermal synthesis and demonstrated excellent NH_3_ sensing performance at elevated temperatures [16].

Metal–organic frameworks (MOFs) have emerged as versatile sacrificial precursors for the synthesis of metal oxides [17]. Owing to their long-range ordered architectures, tunable metal nodes, and uniformly distributed organic linkers, MOFs enable molecular-level mixing of metal species and provide abundant internal porosity [18]. Upon controlled calcination, the organic components are removed while the inorganic nodes are converted into oxide phases, often yielding products with high surface area, and a large density of accessible active sites [19]. Y. Zhao et al. constructed a Zn/Ni bimetallic organic-framework-derived ZnO/NiO heterostructure, in which the Zn:Ni molar ratio of the ZnO/NiO-48 h sample was approximately 4:1, and evaluated its n-propanol sensing performance over 0.2–500 ppm [20]. However, the gas-sensing performance trends strongly depend on the choice of MOF precursors.

Motivated by these merits, in this work, a deliberately selected MOF precursor was used as a sacrificial scaffold to construct NiO/ZnO heterojunctions, aiming to achieve homogeneous phase distribution and tight interfacial coupling between the p-type NiO and n-type ZnO components, which are essential for enhanced charge separation and improved functional performance. The sensing performance of the NiO/ZnO sensor toward n-propanol was systematically evaluated over 2–100 ppm, and selectivity tests were conducted at 100 ppm against several interfering gases. In addition, a random forest algorithm was used to analyze the early-stage response curves, enabling rapid identification of n-propanol concentrations within 7 s.

## 2. Experiment

### 2.1. Preparation of NiO Sample

**Figure 1:**
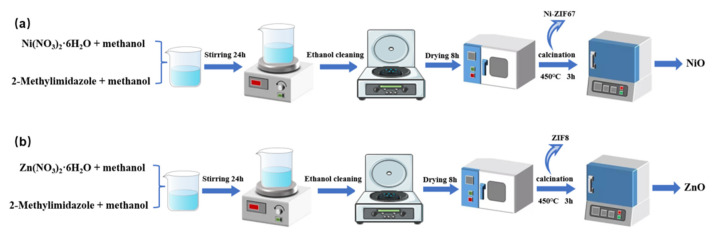
Schematic diagram of the preparation process for (**a**) NiO samples and (**b**) ZnO samples.

As shown in Figure 1a, to prepare NiO using the MOF precursors, Ni-ZIF67 must first be synthesized. We weighed 1 mmol of nickel nitrate hexahydrate (Ni(NO_3_)_2_·6H_2_O, 290.8 mg) and 8 mmol of 2-methylimidazole (2-MeIM, 657 mg). We added 20 mL of anhydrous methanol to the solid reagent. Once the solutions were fully dissolved, we poured the 2-methylimidazole solution into the nickel nitrate hexahydrate solution and stirred the resulting mixture with a magnetic stirrer for 24 h. We washed the stirred mixture three times with ethanol to ensure product purity. Finally, the mixture was dried in an oven at 60 °C for 8 h, forming a yellowish-brown gel, yielding Ni-ZIF67. Subsequently, the sample was calcined in a muffle furnace under air at 450 °C with a heating rate of 5 °C/min for 3 h. The resulting product was a powdered NiO sample.

### 2.2. Preparation of ZnO Sample

As shown in Figure 1b, to prepare ZnO via the MOF precursors, ZIF-8 was first synthesized. Briefly, 1 mmol of zinc nitrate hexahydrate (Zn(NO_3_)_2_·6H_2_O, 297.5 mg) and 8 mmol of 2-methylimidazole (2-MeIM, 657 mg) were each dispersed in 20 mL of anhydrous methanol. After complete dissolution, the 2-MeIM solution was poured into the zinc nitrate hexahydrate solution, and the resulting mixture was magnetically stirred for 24 h. The mixture was then washed with ethanol three times to ensure product purity, followed by drying in an oven at 60 °C for 8 h to form a white gel, which yielded ZIF-8. Subsequently, the dried sample was calcined in a muffle furnace under air at 450 °C with a heating rate of 5 °C/min for 3 h. The final product was a ZnO powder.

### 2.3. Preparation of NiO/ZnO Heterojunction Material

**Figure 2:**
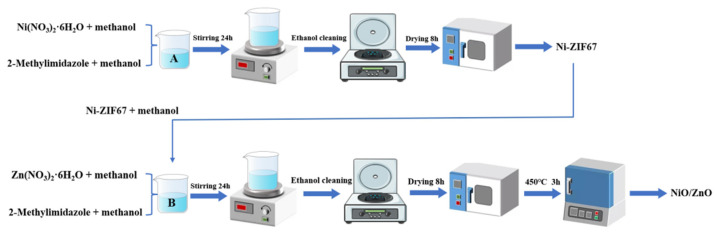
Schematic diagram of the NiO/ZnO heterojunction fabrication process.

As shown in Figure 2, NiO/ZnO samples were prepared using the MOF precursors. The sample preparation procedure is as follows: First, Ni-ZIF67 was prepared by weighing 1 mmol of Ni(NO_3_)_2_·6H_2_O (290.8 mg) and 8 mmol of 2-MeIM (657 mg). We added 20 mL of anhydrous methanol solution to each. After the solutions had fully dissolved, we poured the 2-methylimidazole solution into the nickel nitrate hexahydrate solution, and placed the resulting mixture A in a magnetic stirrer to stir for 24 h. We washed the stirred mixture three times with ethanol to ensure the purity of the product, yielding a yellow precipitate, Ni-ZIF67. Next, we weighed out 1 mmol of Zn(NO_3_)_2_·6H_2_O (297.5 mg) and 8 mmol of 2-MeIM (657 mg). The molar feeding ratio of Ni to Zn precursors was therefore 1:1, which corresponded with the designed NiO:ZnO molar ratio in the final composite after calcination. We added 20 mL of anhydrous methanol to each. After the solutions had fully dissolved, we poured the 2-methylimidazole solution into the zinc nitrate hexahydrate solution, and stirred the resulting mixture in a magnetic stirrer for 24 h. We added 10 mL of anhydrous methanol to the yellow precipitate and sonicated until the precipitate was completely dissolved. Then we poured the mixture into a container and washed it three times with ethanol to ensure the purity of the product. We placed the ethanol-washed mixture in a magnetic stirrer and stirred for 24 h to obtain ZIF-67/ZIF-8 (MOFS precursor). After drying for 8 h, the sample was calcined in a muffle furnace under air at 450 °C with a heating rate of 5 °C/min for 3 h.

### 2.4. Characterization of Gas-Sensitive Materials

In this study, X-ray diffraction (XRD) was employed to systematically characterize the crystal structure, and the phase composition of NiO/ZnO heterojunction samples. The experimental setup utilized an XRD-7000S X-ray diffractometer manufactured by Shimadzu Corporation of Kyoto, Japan. During testing, the 2*θ* scan range was set to 20–80°, with a scan rate of 5°/min. To thoroughly analyze the microstructural characteristics of the synthesized materials, scanning electron microscopy (SEM) was simultaneously employed to observe surface morphology. SEM testing was conducted using a Nova NanoSEM 450 field-emission scanning electron microscope manufactured by FEI Company (Hillsboro, OR, USA). During sample preparation, the samples were first dispersed in high-purity ethanol, then mounted on clean silicon wafers as substrates and securely fixed using a conductive adhesive. The acceleration voltage was set to 18 kV during testing. Furthermore, to accurately determine the elemental chemical states at the NiO/ZnO heterojunction interface and on the ZnO surface, this study employed X-ray photoelectron spectroscopy (XPS) for surface chemical analysis. The instrument used was the ESCALAB™ 250Xi X-ray photoelectron spectrometer manufactured by Thermo Fisher Scientific (Waltham, MA, USA), which provided experimental evidence for the charge transfer and band alignment mechanisms at the heterojunction interface.

### 2.5. Preparation and Tests of Gas-Sensing Devices

**Figure 3:**
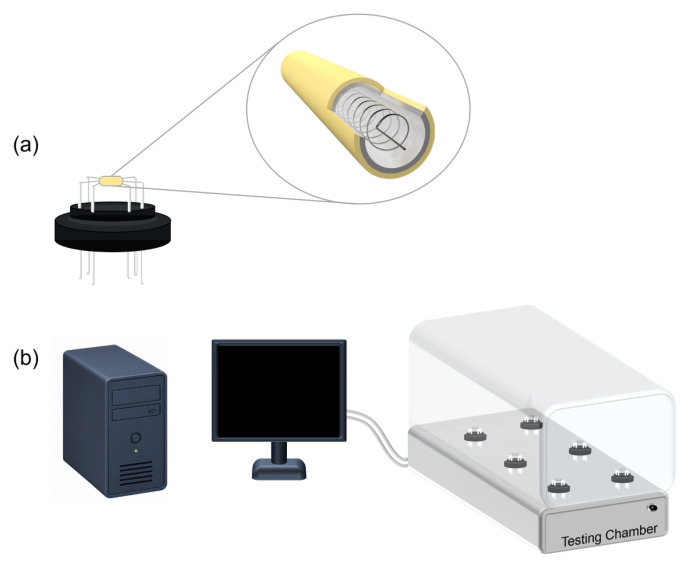
(**a**) Schematic diagram of the sensor. (**b**) Schematic diagram of the CGS-8 Intelligent Gas Sensitivity Analysis System.

The study employed a homogenization coating method to fabricate the gas sensor, as shown in Figure 3a. First, the synthesized material and deionized water were placed in an agate mortar and thoroughly ground to form a uniform paste. Next, the paste was evenly applied to the outer surface of an aluminum oxide ceramic tube using a brush, ensuring complete coverage of the Au electrode regions at both ends of the tube. After coating, the device was placed in a constant-temperature oven at 250 °C for 8 h to dry and age it, thereby improving its stability. The dried and aged sensor was then soldered to a hexagonal base using platinum wire. At the same time, a nickel–chromium alloy heating wire was precisely inserted into the inner cavity of the alumina ceramic tube, and its two ends were soldered to the base.

The gas-sensing performance was evaluated using a CGS-8 Intelligent Gas Sensing Analysis System (Beijing Elite Technology Co., Ltd., Beijing, China), as shown in Figure 3b. Gas-sensing tests were performed using a static gas-mixing method. During the measurement, the sensor was inserted into the test socket and sealed with a well-sealed glass cover. After the system was started, the temperature and relative humidity of the laboratory environment were monitored and recorded in real time. The operating temperature of the gas sensor was adjusted by setting the heating current. The target n-propanol gas was prepared before being introduced into the sensing chamber. Specifically, a 100 ppm n-propanol standard gas was first prepared in a separate gas-preparation vessel by evaporating a calculated volume of liquid n-propanol and allowing sufficient mixing. Lower concentrations of 2, 5, 10, 20, and 50 ppm were then obtained by volume dilution of the 100 ppm standard gas with air. The prepared target gas was introduced into the sensing chamber through the gas inlet for response measurement. Therefore, liquid n-propanol was not directly evaporated inside the sensing chamber during the sensor response measurement. During this process, the system recorded the sensor’s resistance values over time in real time, thereby obtaining key data such as the response values to the target gas and the recovery kinetics curve.

In gas-sensing performance tests, the response value of an n-type semiconductor is defined as the ratio of the sensor’s reference resistance $R_{a}$ in air to its resistance $R_{g}$ in the presence of the target gas, i.e., $Response=R_{a}/R_{g}$ [21]. The response time and recovery time refer to the time required for the sensor’s response to reach 90% of its total change during gas adsorption or desorption, respectively. For each sensing material, three independently fabricated sensors were tested. Each response value was obtained from three repeated measurements, and the error bars in the figures represent the standard deviation of the repeated measurements.

The target VOCs used in this study are in liquid state with a purity of no less than 99.0%. The concentration of the target gas in the gas chamber (C, units: ppm) can be calculated using the following Equation (1):

(1) $$C=\frac{24.45\times P\times \rho \times V_{l}}{M\times V_{g}}$$

Here, P represents the purity of the liquid, ρ represents the density of the liquid (g/mL), $V_{l}$ represents the volume of the liquid sample (μL), $V_{g}$ represents the volume of the gas chamber (20 L), M represents the molecular weight of n-propanol (g/mol), and 24.04 is the conversion constant for the molar volume of a gas (L/mol) under standard conditions (20 °C, 1 atm). The relative humidity of the experimental environment is controlled by an external humidifier and monitored and recorded in real time by a humidity sensor built into the test system to ensure the reproducibility of test conditions and the reliability of data. The uncertainty in the target gas concentration mainly originated from the liquid injection volume for preparing the standard gas, the gas-preparation volume, and the gas-volume transfer during dilution. For the preparation of the 100 ppm standard gas, the liquid injection volume was approximately 6.29 μL. Assuming an operational uncertainty of ±0.1 μL for the microsyringe, the relative error caused by liquid injection was approximately 1.6%. Considering the additional uncertainties from gas volume and transfer operations, the overall relative error of the target gas concentration was estimated to be within approximately 5%.

## 3. Results and Discussion

### 3.1. Characterization of Gas-Sensing Materials

**Figure 4:**
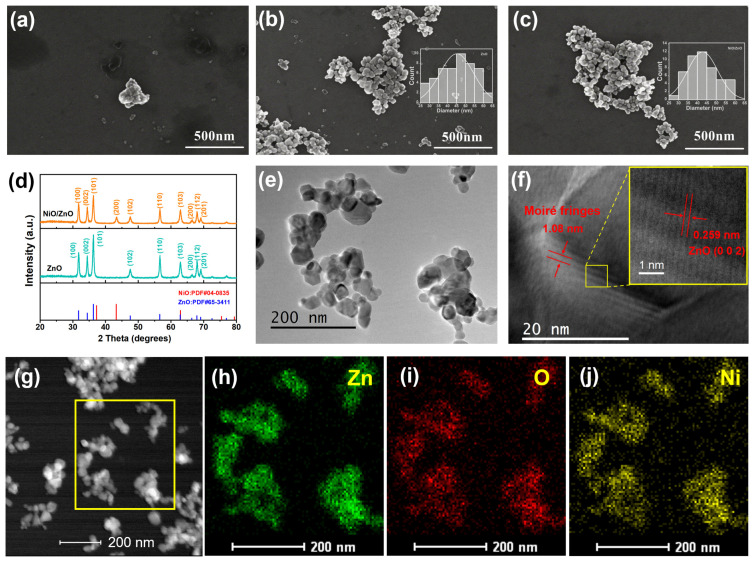
SEM images of (**a**) pure NiO, (**b**) pure ZnO, and (**c**) NiO/ZnO heterojunction. (**d**) The XRD pattern of NiO/ZnO and ZnO. (**e**) TEM image of the NiO/ZnO heterojunction. (**f**) High-resolution TEM image of the NiO/ZnO heterojunction. (**g**) HAADF-STEM image of the NiO/ZnO heterojunction, the boxed region is enlarged in figures (**h**,**j**). Elemental mapping images of the same region showing the distributions of (**h**) Zn, (**i**) O, and (**j**) Ni.

Figure 4a–c shows the SEM images of pure NiO, pure ZnO, and the NiO/ZnO composite. All samples exhibit an agglomerated nanoparticle morphology. The apparent particle sizes of the samples were analyzed from SEM images using ImageJ software (version 1.54f). For pure ZnO and the NiO/ZnO composite, 50 and 49 particles were randomly selected, respectively. The average apparent particle sizes of pure ZnO and the NiO/ZnO composite were 45.2 nm and 42.8 nm, respectively. The corresponding median apparent particle sizes were 46.9 nm and 41.9 nm, respectively. In contrast, pure NiO exhibited a much larger agglomerated particle morphology, with a representative apparent particle size of approximately 195.3 nm based on the SEM image. These values represent SEM-derived apparent particle sizes rather than crystallite sizes.

Figure 4e shows the TEM image of the NiO/ZnO composite. The HRTEM image in Figure 4f presents clear lattice fringes, which can be assigned to the crystalline ZnO phase. No distinct interleaved lattice fringes of ZnO and NiO were observed in HRTEM, whereas obvious Moiré fringes were clearly detected. Moiré fringes originate from the lattice interference of two crystals with different lattice parameters under close interfacial contact and slight orientation deviation, confirming the tight atomic-level interfacial coupling between ZnO and NiO. Quantitative calculations from the Moiré fringes reveal that the lattice spacing of NiO is measured to be 0.209 nm, which is in perfect agreement with the interplanar spacing of the (200) crystal plane of NiO as determined by XRD analysis. Collectively, these results confirm the successful synthesis of the NiO/ZnO heterojunction material.

As shown in Figure 4g–j, the HAADF-STEM image and corresponding elemental mapping results clearly reveal the coexistence and relatively uniform distribution of Zn, Ni, and O in the selected region of the NiO/ZnO sample. These results indicate that ZnO and NiO are effectively combined in the composite material. Together with the XRD results confirming the coexistence of ZnO and NiO crystalline phases and the XPS results showing the shift in Zn 2p peaks toward higher binding energy, as discussed below, the TEM/HAADF-STEM characterization supports the formation of interfacial NiO/ZnO heterojunctions in the composite material.

X-Ray diffraction was used to characterize the crystal structures of ZnO and the NiO/ZnO composite. As shown in Figure 4d, the diffraction patterns exhibit good agreement with the standard PDF cards (ZnO: PDF#65-3411; NiO: PDF#04-0835). The diffraction peaks of the heterojunction sample at 31.7°, 34.4°, 36.3°, 62.6°, and 69.1° correspond to the (100), (002), (101), (103), and (201) crystal planes of ZnO in the hexagonal zincite structure, respectively; while the characteristic peak at 43.3° corresponds to the (200) crystal plane of face-centered cubic NiO [22]. The absence of other impurity peaks or significant peak shifts in the diffraction pattern confirms the stable coexistence of ZnO and NiO in the heterojunction material, providing the basis for the formation of a p-n heterojunction at their interface.

To further evaluate the crystallite characteristics of the samples, the coherent diffraction domain sizes of ZnO and NiO were estimated from the XRD patterns using the Scherrer equation

(2) $$D=\frac{K\cdot \lambda }{\beta \cdot cos\theta }$$

where D is the coherent diffraction domain size, K is the shape factor, taken as 0.9, λ is the wavelength of *Cu Kα* radiation, taken as 0.154 nm, β is the full width at half maximum of the diffraction peak after conversion from degrees to radians, and θ is the Bragg angle corresponding to half of the diffraction peak position 2θ. For the ZnO phase, the ZnO (100), (002), and (101) diffraction peaks were selected for calculation.

The calculated coherent diffraction domain sizes of ZnO in pure ZnO and the NiO/ZnO composite were 22.3 nm and 21.7 nm, respectively. NiO nanoparticles may adhere to the grain boundaries of ZnO, thereby pinning the grain boundaries and suppressing the growth of ZnO grains. Moiré fringes can be observed in the HRTEM image, which originate from the lattice interference between two crystals with different lattice parameters under close interfacial contact and slight orientation deviation. This indicates the presence of close interfacial coupling between ZnO and NiO. Combined with the XRD result showing that the ZnO crystallite size in the composite is smaller than that of pure ZnO, these results further verify the successful construction of the ZnO/NiO heterojunction.

### 3.2. Gas-Sensing Performance

To evaluate the gas-sensing performance of heterojunction materials, this section systematically tested the gas-sensing performance of pure ZnO, pure NiO, and NiO/ZnO heterojunction composites.

**Figure 5:**
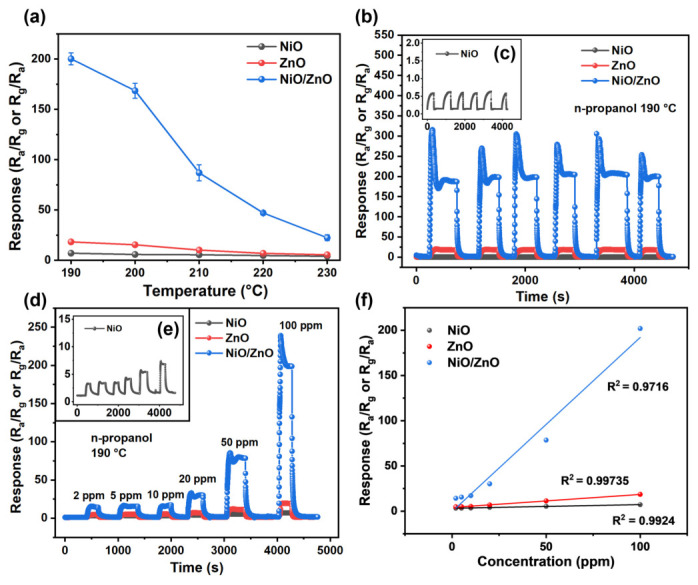
(**a**) Temperature-dependent responses of the sensors toward 100 ppm n-propanol from 190 to 230 °C. (**b**) Cyclic response–recovery curves toward 100 ppm n-propanol at 190 °; (**c**) the enlarged NiO response curve. (**d**) Dynamic response–recovery curves toward 2–100 ppm n-propanol at 190 °C, with (**e**) the enlarged NiO response curve. (**f**) Linear fitting curves of the sensor responses as a function of n-propanol concentration in the range of 2–100 ppm at 190 °C.

Operating temperature is a key parameter affecting gas-sensing performance, as the chemical adsorption and reaction of target gas molecules on the sensor material surface typically require sufficient thermal energy. Figure 5a presents the temperature-response curves of pure ZnO, pure NiO, and the NiO/ZnO heterojunction upon exposure to 100 ppm n-propanol within the temperature range of 190–230 °C. It should be noted that, due to the measurement limitation of the testing system, the lower-temperature data were not presented in this study. The upper resistance measurement limit of the CGS-8 gas-sensing test system used in this work is 500 MΩ. In the preliminary operating-temperature screening experiments, when the operating temperature was lower than 190 °C, the baseline resistance of the NiO/ZnO heterojunction sensor in air exceeded 500 MΩ, which was beyond the reliable measurement range of the instrument. Therefore, stable dynamic response curves could not be obtained below 190 °C. Considering both response performance and practical applicability, 190 °C was selected as the operating temperature for the subsequent gas-sensing tests. As clearly illustrated in Figure 5a, the response value of the NiO/ZnO heterojunction reaches 201.7, which is 33.5 times and 13.8 times higher than those of pure ZnO (6.0) and pure NiO (14.6), respectively. The relatively low response of the pure NiO sensor may be related to its morphological features. As shown in Figure 4a, pure NiO exhibits a more pronounced agglomerated morphology, with an apparent particle size much larger than those of pure ZnO and the NiO/ZnO composite. The larger particle size and stronger aggregation may reduce the effective specific surface area, decrease the number of exposed active sites, and hinder the diffusion of n-propanol molecules within the sensing layer, thereby weakening the gas-sensing response. Therefore, in addition to its intrinsic sensing characteristics, the low response of pure NiO can also be partly attributed to its larger particle size and agglomerated morphology. This confirms that the formation of the heterojunction directly enhances the gas-sensing performance of the material. At 190 °C, the NiO/ZnO sensor exhibits the maximum response value, while the response values of pure ZnO and pure NiO also reach their peaks at this temperature. With increasing temperature, the response values of all materials decrease significantly. This deterioration in sensing performance is likely attributed to the reduced adsorption rate of gas molecules on the material surface at higher operating temperatures [23,24]. Therefore, 190 °C was selected as the optimal operating temperature for the NiO/ZnO sensor in subsequent tests.

Cycle stability is one of the most critical performance indicators for gas sensors in practical applications; if the sensor’s response fluctuates excessively during repeated cycling tests, it cannot achieve accurate, long-term detection of specific gases. To investigate the cycling stability of the materials, this study tested the transient response and recovery characteristics of NiO, ZnO, and NiO/ZnO heterojunction materials in 100 ppm n-propanol gas at an operating temperature of 190 °C. The test results are shown in Figure 5b,c. Clearly, the gas-sensing performance of all three materials did not exhibit significant fluctuations, indicating that the fabricated sensors possess excellent cycling stability. It should be noted that the sharp peak observed at the initial stage of gas exposure in Figure 5b,d is a transient overshoot rather than the final steady-state response. Immediately after n-propanol injection, the local gas concentration near the sensing layer changes rapidly, and n-propanol molecules react quickly with pre-adsorbed oxygen species and active sites on the NiO/ZnO surface. Similar atypical transient response behaviors, including “beak-shaped” responses, have been reported in composite oxide gas sensors and were associated with multiple activation-energy-related states and Fermi-level pinning effects in nanocomposite sensing materials [25].

Figure 5d,e shows the dynamic response curves of NiO, ZnO, and the NiO/ZnO heterojunction at n-propanol concentrations of 2 ppm, 5 ppm, 10 ppm, 20 ppm, 50 ppm, and 100 ppm, i.e., the relationship between response value and n-propanol concentration. The experimental data demonstrate that the resistance of the sensing materials remains stable in air. When n-propanol gas is introduced into the sealed test chamber, the resistance of the sensing materials decreases until it reaches a stable value; after opening the test chamber and purging the n-propanol gas, the resistance of the sensing materials recovers to its initial stable value in air. This resistance-decreasing behavior indicates that the NiO/ZnO heterojunction exhibits n-type sensing behavior toward reducing n-propanol gas. As the n-propanol gas concentration increases, the resistance of the NiO/ZnO heterojunction to n-propanol shows a gradual downward trend.

**Figure 6:**
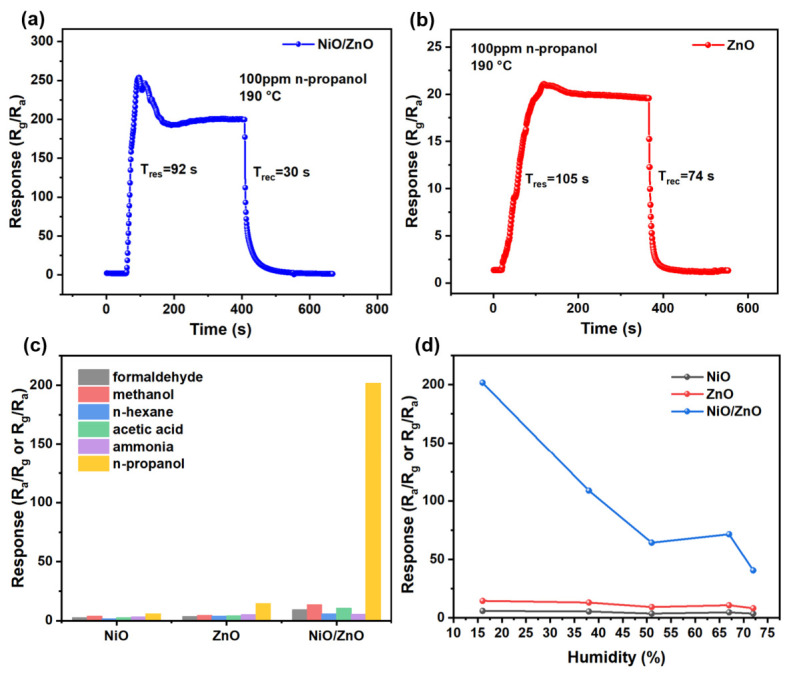
Response/recovery time of (**a**) NiO/ZnO heterojunction material, and (**b**) ZnO to 100 ppm n-propanol at 190 °C. (**c**) Gas-sensing responses of NiO, ZnO, and NiO/ZnO to 100 ppm n-propanol, n-hexane, formaldehyde, acetic acid, ammonia, and methanol. (**d**) Relative humidity stability.

Figure 5f presents the concentration–response fitting curves of pure NiO, pure ZnO, and the NiO/ZnO heterojunction toward n-propanol at 190 °C. Within the investigated concentration range, the response values of pure NiO and pure ZnO increase approximately linearly with increasing n-propanol concentration. However, the response of the NiO/ZnO heterojunction exhibits a lower linear dependence on concentration. Figure 6a,b present the gas-sensing response and recovery curves of the NiO/ZnO heterojunction material and pure ZnO, respectively, for 100 ppm n-propanol at an operating temperature of 190 °C. For the NiO/ZnO heterojunction material (as shown in Figure 6a), the response value rises rapidly upon introduction of the target gas, with a response time of only 92 s. After ceasing the introduction of n-propanol, the material rapidly returns to its initial state, with a recovery time of 30 s, demonstrating highly efficient response–recovery kinetics. Compared to pure ZnO (response time: 105 s; recovery time: 74 s), these times were reduced by 13 s and 44 s.

Figure 6c shows a comparison of the gas-sensing responses of three materials—NiO/ZnO, NiO, and ZnO—to six 100 ppm target gases: n-propanol, n-hexane, formaldehyde, acetic acid, ammonia, and methanol. The test results indicate that the NiO/ZnO heterojunction material exhibits extremely strong response specificity toward n-propanol, with a response value as high as approximately 200, far exceeding the response values for n-hexane, formaldehyde, acetic acid, ammonia, and methanol. The responses of pure NiO and ZnO to all gases remained at extremely low levels. The response values for the remaining five interfering gases are all low, showing a clear order-of-magnitude difference compared to n-propanol, which fully demonstrates that the NiO/ZnO composite material possesses excellent detection selectivity for n-propanol. The selectivity of metal oxide gas-sensing materials is generally influenced by several factors, including gas adsorption, surface active sites, oxygen vacancies, and surface reaction kinetics. Although other reducing gases can also react with adsorbed oxygen species, their adsorption and reaction behaviors on the ZnO/NiO surface may be different. However, the detailed selectivity mechanism still requires further investigation, and DFT calculations and in situ spectroscopic characterizations will be carried out in our future work for further verification. Figure 6d shows the response values of NiO, ZnO, and NiO/ZnO as a function of humidity. The response values of all three materials decrease gradually as humidity increases, dropping sharply in the 10–50% humidity range; once humidity reaches the intermediate level of 50%, the rate of decline slows, though the response values remain lower than those observed in the 10–50% humidity range. The response value of NiO/ZnO is significantly higher than that of pure NiO and pure ZnO. The reasons for these changes in response values may be explained as follows: when water vapor enters the test chamber, adsorbed oxygen reacts with water molecules to form hydroxyl radicals (as shown in the reaction equation below):

(3) $$H_{2}O_{\left(g\right)}+O_{\left(ads\right)}^{-}\to 2{OH}_{\left(ads\right)}+e^{-}$$

This process impedes the sensor’s response to n-propanol in two ways: the adsorption of water molecules on the material’s surface directly occupies oxygen active sites, hindering the reaction between n-propanol and chemisorbed oxygen [26,27]; water molecules consume surface-adsorbed oxygen, reducing the concentration of oxygen species on the material’s surface, which further weakens the response of the gas-sensing reaction and ultimately leads to a decrease in the sensor’s response to n-propanol. The NiO/ZnO heterojunction sensor exhibits a high response of 201.7 toward 100 ppm n-propanol at a relatively low operating temperature of 190 °C. Compared with other metal oxide-based sensors shown in Table 1, the as-fabricated sensor demonstrates superior gas-sensing performance, including fast response and a moderate working temperature.

As shown in Table 1, the present NiO/ZnO sensor exhibits competitive n-propanol sensing performance compared with previously reported metal oxide semiconductor sensors, especially in terms of operating temperature, response value, and response/recovery characteristics.

Humidity is an important factor affecting the practical application of metal oxide semiconductor gas sensors. However, not all the sensors summarized in Table 1 reported humidity-dependent sensing data, making a direct comparison difficult. In general, water molecules may compete with oxygen species and n-propanol molecules for surface active sites, thereby reducing the sensing response. Therefore, humidity tolerance is an important consideration for the practical application of n-propanol sensors.

### 3.3. Mechanism of Gas-Sensing

**Figure 7:**
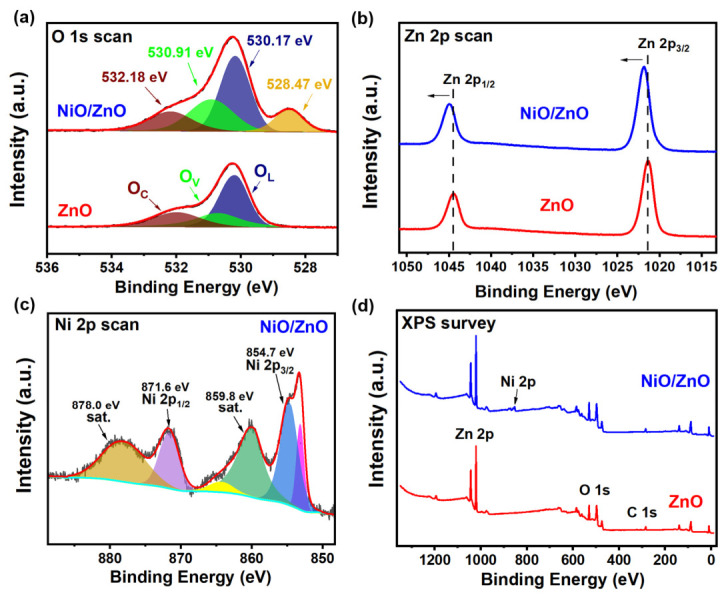
(**a**) O 1s spectra of NiO/ZnO and ZnO, (**b**) Zn 2p spectra of NiO/ZnO and ZnO, (**c**) Ni 2p spectrum of NiO/ZnO, and (**d**) XPS survey spectra of NiO/ZnO and ZnO.

Given that NiO/ZnO heterojunction materials exhibit excellent gas-sensing performance and that the surface state of the material significantly influences gas-sensing characteristics, it is crucial to analyze the surface state of the material’s components. Figure 7a,b present the chemical composition and valence states of ZnO and NiO/ZnO as determined by XPS analysis. The binding energy shifts were calibrated using the C 1s peak (284.8 eV) as a reference.

Figure 7a shows the O 1s spectrum; the high-intensity peaks at 530.17 eV and 528.91 eV correspond to lattice oxygen (O_L_) in ZnO and NiO, respectively. Since the chemical environment of oxygen in NiO/ZnO differs from that in pure ZnO and NiO, this results in certain shifts in the characteristic peaks [40]. The O 1s peak at 530.9 eV corresponds to oxygen vacancies (O_V_), while the broad peak at 532.1 eV is associated with chemically adsorbed oxygen (O_C_) [26]. Additionally, calculations indicate that the ratios of O_V_ and O_C_ in pure ZnO samples are 14.71% and 14.24%, respectively. In NiO/ZnO, due to the formation of a p-n heterojunction, the ratios of O_V_ and O_C_ increase to 15.03% and 19.32%, respectively. This provides more active sites for the target gas, n-propanol, during the sensing process, thereby further enhancing the sensing performance of the ZnO/NiO sensor.

The XPS spectrum of Zn 2p in ZnO (Figure 7b) exhibits a doublet with peak positions at 1044.6 and 1021.5 eV, attributed to Zn 2p_1/2_ and Zn 2p_3/2_, respectively. Notably, after coupling with NiO, the Zn 2p peaks in the NiO/ZnO heterostructure shift by 0.5 eV toward higher binding energy [41]. This shift indicates a decrease in the electron density around Zn sites, suggesting electron transfer from ZnO to NiO and supporting the formation of interfacial NiO/ZnO heterojunctions. Compared with single ZnO or NiO, the superior sensing performance of the NiO/ZnO heterostructure is mainly attributed to the synergistic effect of interfacial p-n heterojunction modulation on the enriched surface active site. The contact between n-type ZnO and p-type NiO forms an interfacial depletion region and built-in electric field, making the resistance more sensitive to surface gas reactions. Meanwhile, the increased oxygen vacancies and chemisorbed oxygen species provide more reaction sites for n-propanol molecules. Therefore, the NiO/ZnO heterostructure can amplify the resistance variation during gas adsorption and oxidation, leading to a higher response than single ZnO and NiO. The Ni 2p spectrum of the NiO/ZnO composite can be divided into Ni 2p_3/2_, Ni 2p_1/2_ and their corresponding satellite features. The fitted peaks around 853–855 eV are assigned to Ni^2+^ species in a Ni-O coordination environment, while the peaks around 860 and 878 eV are attributed to the shake-up satellite features of Ni^2+^, which are typical characteristics of divalent nickel. Therefore, Ni mainly exists as Ni^2+^ in the NiO/ZnO composite. In the XPS survey spectrum of Figure 7d, in addition to the Zn and O signals, a Ni 2p signal can be observed in the binding energy range of approximately 850–890 eV, confirming the presence of Ni in the NiO/ZnO composite. Based on the above results, the enhanced response of the NiO/ZnO heterojunction toward n-propanol is discussed from two main perspectives:

1. The p-n heterostructure between ZnO and NiO;
2. Oxygen vacancies existing at the interface.

Typically, the response of oxide semiconductor sensors manifests as a change in resistance. In n-type ZnO semiconductors, electrons serve as the primary charge carriers, whereas in p-type NiO semiconductors, holes serve as the primary charge carriers.

When an n-type pure ZnO gas sensor is exposed to air, oxygen molecules adsorb onto the material surface and capture electrons from the conduction band to form chemisorbed oxygen, creating an electron depletion layer on the surface. When exposed to n-propanol, the n-propanol reacts with the adsorbed oxygen, causing the depletion layer to become thinner and the sensor resistance to decrease, whereas the response behavior of a p-type semiconductor sensor is exactly the opposite. The formation process of the oxygen-adsorbed species can be described by the following equation [42,43,44].

(4) $$O_{2(gas)}\to {\;O}_{2(ads)}$$

(5) $$O_{2(ads)}+e^{-}\to O_{2(ads)}^{-}$$

(6) $$O_{2(ads)}^{-}+e^{-}\to {2O}_{\left(ads\right)}^{-}$$

(7) $$C_{3}H_{7}OH\left(ads\right)+O^{-}\left(ads\right)\to \;C_{3}H_{6}O+H_{2}O+e^{-}$$

**Figure 8:**
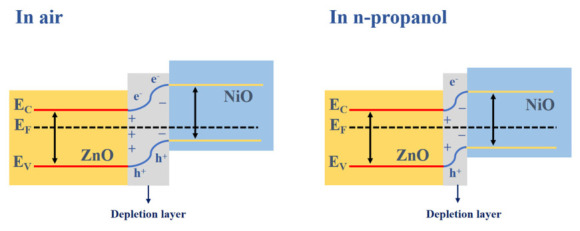
Mechanism diagram of the NiO/ZnO p-n heterojunction.

However, the gas-sensing mechanism of NiO/ZnO differs slightly from that of pure ZnO. When the two metal oxides come into contact, because the Fermi level of NiO is lower than that of ZnO, electrons move from ZnO to NiO, until the system reaches equilibrium (Figure 8) [45]. At equilibrium, an electron depletion layer forms at the ZnO/NiO interface, which acts as a heterojunction barrier between the two materials. In air, oxygen molecules adsorb onto the NiO/ZnO surface and capture free electrons from ZnO to form oxygen species (O_2_^−^, O^−^). This thickens the depletion layer, resulting in the relatively high resistance of the NiO/ZnO heterojunction sensor. Upon exposure to the reducing gas n-propanol, the propanol molecules react with the oxygen species adsorbed on the surface, releasing the captured electrons. The charge transfer efficiency is improved, and the electrons/holes generated by the gas reaction can rapidly participate in conduction, resulting in a faster response speed. Second, more oxygen vacancies exist at the interface, providing more sites for gas adsorption and reaction.

### 3.4. Rapid Identification

**Figure 9:**
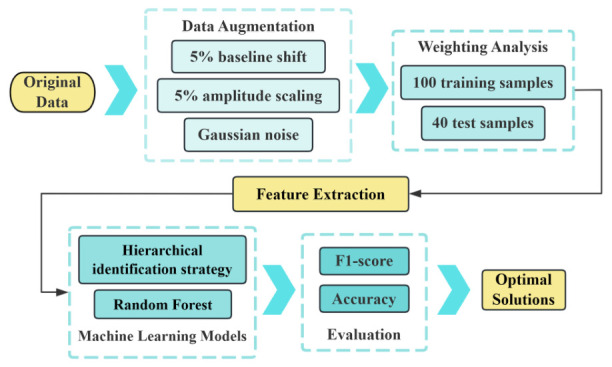
Schematic description of the workflow diagram.

To enable rapid identification of n-propanol concentration, this study conducted machine learning training based on data showing the temporal variation in NiO–ZnO gas-sensitive sensor responses at different gas concentrations. The rapid recognition model developed in this study was established and evaluated under well-defined experimental conditions, including an operating temperature of 190 °C, a relative humidity of 35% RH, n-propanol as the target analyte, and a concentration range of 2–100 ppm. The model was implemented in Python 3.11.5 using PyCharm Community Edition 2023.2.3 as the integrated development environment, with pandas 2.0.3, NumPy 1.24.3, and scikit-learn 1.3.0 employed for data processing, numerical computation, and model construction. The model was developed and tested at a fixed humidity of 35% RH. Therefore, it is currently suitable only for this fixed humidity condition, and different humidity levels will be considered in future work. Since each concentration dataset contained only a single data point, the limited data volume made it impossible to divide the data into training and testing sets. Therefore, this study introduced multi-source random disturbances to the original dataset, including ±5% baseline drift, ±5% amplitude scaling, and Gaussian noise with a standard deviation of 0.025, thereby artificially creating disturbance conditions. Random perturbations were introduced into each raw response curve and resampled to generate 100 training samples and 40 test samples, respectively, thereby approximating the construction of reliable training and test sets under limited experimental data conditions. The overall workflow of the rapid identification method is shown in Figure 9.

To avoid the model’s reliance on the full response curve, the eigenvalues constructed by this method include the current response value, the first-order derivative (which characterizes the rate of change), and the second-order derivative (which characterizes the trend of change). The eigenvalue $x_{t}$ at time t is expressed by the following formula:

(8) $$x_{t}=\;\left[y_{t},\;y_{t}-\;y_{t-1},\;y_{t}-\;2y_{t-1}+\;y_{t-2}\right]$$

Here, $y_{t}$ represents the sensor response value at time t, $y_{t}-\;y_{t-1}$ represents the first-order difference, and $y_{t}-\;2y_{t-1}+\;y_{t-2}$ represents the second-order difference, which corresponds to the slope and acceleration of the data sequence, respectively. Since the model exhibits similar response curve shapes at 2, 5, and 10 ppm, a hierarchical classification strategy was adopted in the model design. First, samples were categorized into low-concentration (≤10 ppm) and high-concentration (≥20 ppm) groups, followed by further subdivision within each group. This approach alleviates the classification difficulties caused by the similarity of response curves in the low-concentration range. Random forest models were employed at each stage to achieve superior nonlinear representation capabilities and robustness against interference.

**Figure 10:**
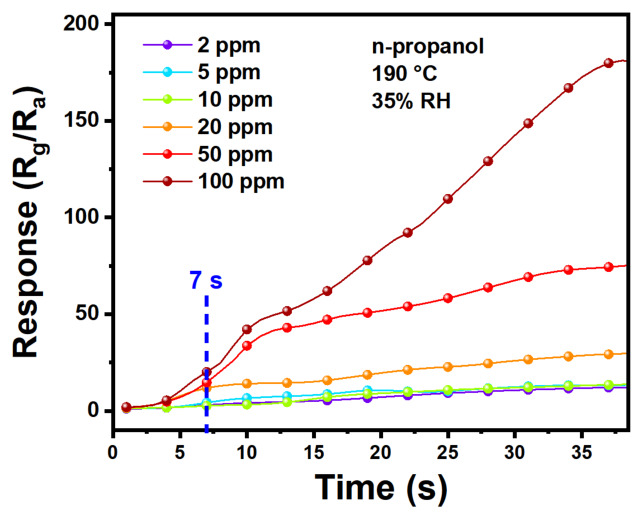
Response curves of the NiO/ZnO sensor at different concentrations of n-propanol.

Model performance was evaluated based on prediction accuracy. As shown in Table 2, during the initial response phase (up to approximately 4 s), the differences between concentration signals were small and significantly affected by noise, making it difficult for the model to distinguish them effectively. As time progressed, high-concentration samples rapidly formed a distinguishable pattern, while some overlap remained in the low-concentration range. When the time reaches approximately 7 s, the accuracy for low concentrations appears to stabilize, indicating that the model has achieved a balance between discrimination capability and response time at this point. Thereafter, as the signals gradually approach saturation, further performance improvements are limited. Therefore, it can be concluded that 7 s represents the minimum detection time required for this sensor system to achieve reliable gas concentration discrimination, well before the gas reaches its response peak (as shown in Figure 10), enabling rapid initial response. The detailed prediction accuracy at different response times is listed in Table 3.

## 4. Conclusions

In this study, the NiO/ZnO heterojunction material prepared via the construction of MOFs exhibited a sensing response value of 201.7 for n-propanol gas, which is 33.5 times and 13.8 times higher than that of the other two materials (ZnO: 6 and NiO: 14.6) at 190 °C for 100 ppm n-propanol. The sensor exhibits n-type sensing behavior toward reducing n-propanol gas and shows good selectivity, with a good linear fitting relationship between response and concentration (R^2^ = 0.97168). Finally, to enable rapid identification of n-propanol gas concentrations, this study developed and validated a method suitable for rapid identification of n-propanol gas concentrations by training and predicting the response curves using a random forest algorithm, achieving identification of n-propanol gas at different concentrations within 7 s.

## Figures and Tables

**Table 1:** Comparative analysis of the sensor response performance in this study and the characteristics of similar sensors in other studies.

No.	Sample Composition	Temperature (°C)	n-Propanol Concentration (ppm)	Response	Reference
1	ZnO/NiO heterojunction	275	500	280.2	[20]
2	p-TiO_2_ NP/n-ZnO NS	RT	100	68	[28]
3	5% SnO_2_-ZnO	200	20	128	[29]
4	3% Ho-doped ZnO	140	100	341	[30]
5	chromosome-like HoFeO_3_	120	100	120.5	[31]
6	LaFeO_3_-La_2_O_3_	100	100	102.2	[32]
7	LaFeO_3_-Fe_2_O_3_	100	100	94.9	[32]
8	3% NiO-In_2_O_3_	275	100	60	[33]
9	4% rGO-In_2_O_3_-4% Ce	200	100	127.3	[34]
10	ZnSnO_3_/ZnO	220	100	14.7	[13]
11	Zn_2_SnO_4_/SnO_2_	270	100	44	[14]
12	Hollow CuO fibers	200	100	4.66	[35]
13	La[Fe(CN)_6_]·5H_2_O-derived LaFeO_3_	100	100	19.2	[36]
14	1 mol% co-modified ZnO nanorods	250	100	491	[37]
15	Hollow NiFe_2_O_4_ hexagonal bipyramids	120	200	32.19	[38]
16	NiO/WO_3_ heterojunction	RT	30	4.8	[39]
17	**NiO/ZnO heterojunction**	**190**	**100**	**201.7**	**This Work**

**Table 2:** Time-dependent response data for NiO–ZnO gas sensors.

Time (s)	2 ppm	5 ppm	10 ppm	20 ppm	50 ppm	100 ppm
1	1.125	1.332	1.374	1.58	1.812	1.957
2	1.179	1.35	1.421	1.951	2.23	2.181
3	1.312	1.435	1.525	3.051	3.113	2.939
4	1.582	1.708	1.716	5.042	4.708	5.416
5	2.004	2.277	1.984	7.702	7.046	10.216
6	2.495	3.186	2.3	10.182	10.182	15.74
7	2.988	4.27	2.599	11.906	14.713	20.077
…	…	…	…	…	…	…

**Table 3:** Data on the change in prediction accuracy over time.

Time (s)	Accuracy	2 ppm	5 ppm	10 ppm	20 ppm	50 ppm	100 ppm
3	91.70%	100.00%	72.50%	82.50%	100.00%	95.00%	100.00%
4	91.20%	90.00%	67.50%	90.00%	100.00%	100.00%	100.00%
5	98.80%	95.00%	100.00%	97.50%	100.00%	100.00%	100.00%
6	99.60%	97.50%	100.00%	100.00%	100.00%	100.00%	100.00%
7	100.00%	100.00%	100.00%	100.00%	100.00%	100.00%	100.00%

## Data Availability

The data presented in this study are available on request from the corresponding author.
